# Innovative talent agglomeration, spatial spillover effects and regional innovation performance—Analyzing the threshold effect of government support

**DOI:** 10.1371/journal.pone.0311672

**Published:** 2024-10-31

**Authors:** Li Yan, Sun Fan, Li Mengyu

**Affiliations:** 1 School of Business Administration, Henan University of Economics and Law, Zhengzhou, China; 2 The Postdoctoral Research Station of the School of Business, Henan University, Kaifeng, China; 3 School of Management and Economics, North China University of Water Resources and Electric Power, Zhengzhou, China; Instituto Tecnologico Autonomo de Mexico, MEXICO

## Abstract

As the primary source of innovation in science and technology, the gathering of creative talent plays a significant role in fostering regional innovation and providing the impetus needed to realize the Chinese people’s aspiration of a great national rejuvenation. Using panel data from 31 provinces over a 12-year period between 2009 and 2020, the spatial Durbin model is constructed to examine the spatial spillover impact of talent agglomeration on the advancement of regional innovation performance, and the panel threshold model is identified and set up to consider whether the nonlinear effect between talent agglomeration and regional achievements in innovation is significant. The analysis demonstrates that: talent pooling has a non-linear effect on the level of innovation performance development, within a certain scale, talent pooling produces an increasing marginal contribution to innovation performance, but after exceeding the limit, it produces a diminishing marginal contribution; the double threshold effect of talent pooling on regional innovation performance is more significant, and government support as a moderating variable confirms that there is a structural mutation between talent pooling and innovation capability. The structural mutation, within a certain threshold range, plays the role of efficient promotion, providing reference for improving regional innovation level.

## 1. Introduction

In today’s fiercely competitive global economic landscape, the concentration of innovative talent has emerged as a pivotal strategy for nations aiming to foster economic growth and regional advancement [1]. China, in particular, has witnessed a profound transformation in its economic structure alongside rapid advancements in science and technology, elevating the significance of innovative talent. These individuals not only drive scientific and technological progress but also serve as crucial catalysts for enhancing regional innovation capabilities. Through efforts to attract and nurture high-caliber talent, China has significantly accelerated scientific and technological innovation and industrial upgrading, while also assuming a more prominent role within the global innovation ecosystem [2]. Consequently, the exploration and facilitation of innovative talent clusters and the enhancement of regional innovation capabilities have become integral components of China’s strategic development agenda.

However, the relationship between innovation talent concentration and regional innovation performance is not simple. In some cases, when the innovative talents in the region cannot form a sufficient scale or the gathering speed is too slow, it may lead to the shortage of human resources in the region, thus hindering the development of the local economy to a certain extent [3]. On the contrary, if innovative talents gather too fast or too many in a certain region, it may lead to excessive competition and unreasonable allocation of human resources, which will limit the maximization of innovation benefits and hinder the improvement of innovation performance in this region and neighboring regions [4]. Therefore, in order to achieve the goal of independent and talent-led development of science and technology, it is necessary to deeply explore whether there is a complex interaction and spatial effect between the concentration of innovative talents and regional innovation performance.

The existing literature offers insights into this issue from an economic perspective. Initially, scholars have employed various metrics such as the location entropy index, fuzzy fixed set, and talent density to assess innovative talents [5, 6]. Their findings indicate a notable concentration of innovative talent in China, notably denser in the southeast and sparser in the northwest regions [7, 8]. Furthermore, researchers have utilized concepts like knowledge management and spatial spillover effects to elucidate how the diffusion of knowledge from innovative talents enhances technological innovation levels in neighboring regions, thereby contributing to regional economic advancement [9, 10]. Additionally, some scholars argue that China continues to face challenges regarding a shortage of high-level talents, which poses constraints on the driving force of innovation necessary for achieving high-quality economic development [11]. Despite these observations, significant developmental potential and opportunities remain untapped. However, current studies often fall short in thoroughly exploring the underlying reasons behind the geographical disparities in China’s innovative talent distribution and proposing effective strategies for improvement.

Secondly, the relationship between innovation talent concentration and regional innovation performance is complicated. Chen et al. (2012) found that, taking knowledge talents in a region as the research object, knowledge innovation output plays a supporting role in improving regional innovation performance [12]. Palious and Wang (1996) pointed out that the collection, dissemination and application of knowledge brought by intra-regional talent exchange is an important source of external knowledge sharing and innovation performance improvement [13]. Based on urban panel data, scholars Cui Xiangmin et al. (2022) studied the impact of the agglomeration of innovative talents and high-quality economic development, and confirmed that the aggregation of innovative human capital within a region can improve innovation efficiency and stimulate economic vitality [14]. However, other scholars have found that the relationship between innovation talent concentration and regional innovation output is not necessarily linear. Scholars Boldrin (2003) and Chen Ying et al. (2019) pointed out that human capital may be mismatched under certain conditions, which will have a negative effect on innovation performance [15, 16]. Hu Bei (2013) believes that excessive concentration of human capital may lead to waste of innovative talent resources and low efficiency of resource allocation [17]. The research of Ma Ru et al. (2019) confirmed that the proportion of scientific and technological talents in human capital in China is still low, and regional imbalance ratio is still an important factor hindering innovation performance and output, so the potential of talent agglomeration and release needs to be stimulated [18]. Existing studies have different views on the relationship between innovation talent agglomeration and regional innovation performance, but they have not considered the possible threshold effect between the two.

Finally, there may be a spatial effect between innovation talent agglomeration and regional innovation performance. Krugman (1991) proved in his research that the agglomeration of talent capital would lead to the spatial agglomeration within the industry and the improvement of regional innovation output in the same way as physical capital [19]. Guo Jinhua et al. (2020) measured the spatial effect of the agglomeration of innovative talents on the growth of total factor productivity and confirmed the impact of the agglomeration of innovative talents in scientific research institutions on regional output efficiency [20]. Han Jun et al. (2022) verified the significant spatial spillover effect of industrial structure adjustment and showed the inhibitory effect on regional innovation performance within the scope [21]. However, in general, there is still a lack of specific discussion on the impact of innovation talent agglomeration on regional innovation performance and the spatial spillover effect. The impact of talent agglomeration differences on innovation performance growth, which only focus on geographical agglomeration and ignore the heterogeneity of innovation entities, still needs to be further developed to make up for the spatial effect brought by innovation talent agglomeration in a smaller geographical scale. Pay attention to the regulatory effect of the government’s macro-control on regional innovation performance under the condition of talent agglomeration, and jointly affect regional innovation activities.

Therefore, this paper selects panel data of 31 provinces (municipalities and districts) in China from 2009 to 2020 (sample data of Hong Kong, Macao Special Administrative Region and Taiwan region are not covered for the time being) for analysis, and empirically explores the impact of innovation talent agglomeration on regional innovation performance. This paper may make the following three contributions: First, based on the provincial data of China, the paper constructs the performance indicators of innovation talent agglomeration and regional innovation, and analyzes the differences and discusses the relevant influencing factors, so as to intuitively show the development of China in these two aspects from the macro level, so as to avoid the insufficiently measured enterprise data at the micro level; Secondly, the spatial effect between innovation talent agglomeration and regional innovation performance is analyzed, which expands the application boundary of innovation talent resource allocation theory, and provides reference for rational allocation of intra-regional innovation talent agglomeration and optimization of inter-regional resource allocation. Third, measure the threshold recognition and effect of marketization degree and government support, so as to provide decision-making reference for grasping institutional advantages in the region and agglomeration of spatial collaborative innovation talent resources, and also provide certain explanations for existing studies on whether these two kinds of effects promote or inhibit.

## 2. Theoretical analysis and research hypothesis

**Fig 1:**
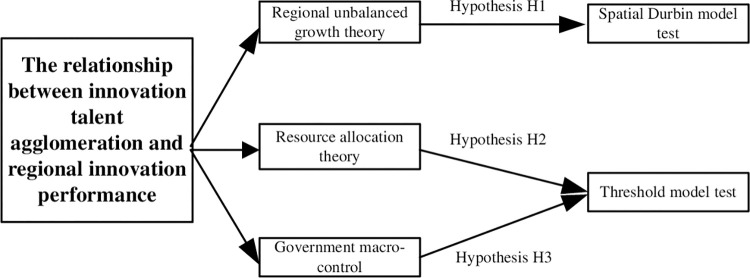
Research frame diagram.

As a leading subject in the industry with innovative consciousness, innovative spirit, innovative thinking, innovative knowledge and innovative ability, innovative talents have more tacit knowledge. Therefore, the clustering phenomenon of innovative talents, as the main body of knowledge innovation and spillover, is bound to bring the effect of knowledge spillover within the region, and therefore bring the synergistic interaction effect of knowledge sharing and dissemination between them. Based on the theory of regional unbalanced growth [22], the development of each region depends on different industrial clusters, and the regions producing innovation clusters will form economic growth poles, which will bring about the aggregation of innovative enterprises and the introduction of talents, form scale effects and innovation effects, and facilitate the knowledge spillover of surrounding regions. On the one hand, the gathering of innovative talents in a specific region presents a phenomenon of a small number into a large number of talents, which helps to break the space-time barrier of knowledge transmission, form a situation of innovation cooperation, efficient interaction, risk sharing and benefit sharing, and provide a basic guarantee for innovation entities to improve regional innovation performance. On the other hand, when the cluster of innovative talents reaches a certain scale, it will help to form a regional science and technology innovation center, promote the allocation of surrounding innovation resources, and generate spillover effects through spatial proximity. For example, the cluster of innovative talents will generate inter-regional knowledge spillover through academic exchanges and project cooperation, which is conducive to the improvement of innovation performance within the region. Based on the above analysis, the following research hypotheses are proposed (Fig 1):

H1: The agglomeration of regional innovative talents is conducive to the growth of regional innovation performance and the spatial spillover effect is obvious;

In the theory of resource allocation [23], achieving optimal allocation of scarce resources requires integrating limited innovative talent across different regions. Furthermore, in the context of a market economy and proactive governmental regulation, institutional advantages should be fully leveraged to optimize the allocation of innovative human resources [24]. Within a market-oriented framework, the aggregation and allocation of innovative talents aim to maximize profitability and efficiency. Regions with superior marginal returns enjoy advantages in attracting and retaining talent. As market mechanisms mature, they facilitate the free flow of talent and unlock the latent potential of innovative individuals. Market-based matchmaking reduces costs associated with information asymmetry among innovation stakeholders, thereby fostering innovation vigor and boosting innovation output. Despite advocating for a "strong government + strong market" dual-strength system in the era of reform and opening up, practical challenges persist. Enterprises affiliated with the government tend to possess greater financial and talent resources, disadvantaging other players in the innovation ecosystem. Moreover, burnout among non-production innovators and a declining desire for entrepreneurial activity further dampen enterprise R&D efforts, hindering regional innovation performance. Based on the aforementioned analysis, the following research hypothesis is proposed (Fig 1):

H2: The process of marketization significantly impacts the aggregation of innovative talents, thereby influencing regional innovation performance positively.

Additionally, the government, as an essential participant, exercises macro-control over resource allocation, including policy formulation and subsidies, to proactively influence governance [25]. This includes providing subsidies or tax incentives tailored to different types of innovation entities and projects. For instance, scientific research institutions—such as universities and specialized research centers—engaged in foundational research and knowledge development, require financial subsidies and governmental funding to offer tangible incentives for attracting innovative talents to the region. These incentives may include salaries, grants, and rewards, aimed at boosting the efforts of innovative talents, enhancing their enthusiasm, and fostering career satisfaction. This, in turn, promotes regional innovation performance. Government support provides an important decision-making reference for the rational allocation of innovation talent agglomeration in the region, optimising resource allocation, grasping the institutional advantages within the region, and spatial synergism in the agglomeration of innovation talents and resources. Moreover, as a strategy to encourage independent innovation within enterprises, government expenditure on research and development is anticipated to significantly bolster R&D activities among small and medium-sized enterprises. This investment is expected to catalyze talent recruitment policies and advance innovation initiatives within these enterprises, thereby enhancing regional innovation performance. Based on this analysis, the following research hypothesis is proposed (Fig 1):

H3: Government support enhances the impact of innovation talent aggregation on regional innovation performance.

## 3. Study design

### 3.1. Variable selection and variable description

The explained variable is Regional Innovation Performance (RIP): from the standpoint of innovation output, the measure of intermediate output mainly includes the quantity of applications for invention patents as well as the quantity of invention patents granted. This study incorporates Jing Li et al.’s research [26]. The authorized quantity of invention patents is used to determine the output of regional achievements in innovation.

The key explanatory variable is Innovation talent Cluster (ITA). In this paper, we measure the indicators of innovative talent agglomeration with the help of location entropy index. References from Wang Meng et al and Zhang Tingting et al [27, 28]. The reality of economic development level, environmental regulation intensity, and technological innovation ability among different regions are in large differences [29]. The knowledge-intensive occupations of innovative talents are defined as the super innovation core, and R&D is selected as the main indicator to measure innovation with more practical application value. Referring to the practice of Guo Jinhua and Guo Shufen [20], the paper specifically uses "the ratio of full-time equivalent of R&D personnel in a region to the total number of employees in the region and the ratio of national R&D personnel in the total number of employees" to measure the concentration level of innovative talents in each region

$$agg_{it}=(rdn_{it}/n_{it}){\left(RDN_{it}/N\right)}_{it}$$ (1)

*rdn* represents the regional R&D personnel input, RDN represents the national R&D personnel input, *n* represents the entire number of workers in the area at year’s conclusion, and *N* represents the entire number of employees at year’s conclusion in the country.

Threshold variables consider the degree of marketization (DOM): the marketization process of a region, determined by "the ratio of each region’s fixed asset investment, employment, and non-state economy’s production value weighted average"; the intensity of government support (IGS): the government’s regulation is to take note of the R&D status of the main innovation actors to give support to the intensity of the support. Therefore, it is interpreted by "the percentage of R&D spending on all government spending".

The control variables adopted in this paper are as follows:

First, the level of informatization (LOI), which is measured by the Internet penetration rate. The higher the level of network informatization of a city, the more conducive it is to carry out innovative activities, reduce information asymmetry, and lay the information service foundation for improving the level of economic development;

Second, Foreign investment (TFI) index: the proportion of foreign direct investment in GDP of each province is measured and converted according to the average exchange rate of RMB against US dollar in the current year;

Third, urbanization rate (LOU): the level of urbanization is measured by the ratio of urban population to total population. Urbanization can transfer factors from the low-productivity agricultural sector to the high-productivity sector, and promote high-quality economic development by improving the effective allocation of resources.

Fourth, the level of transport infrastructure (TIL), which is measured by the number of road miles per square kilometer. The better the transport infrastructure, the more attractive it is to innovative talents and industries, and can improve the quality of economic development.

Fifth, investment scale (TIS): measured by the scale of state-owned economic fixed asset investment in each province and the proportion of total social fixed asset investment;

Sixth, population density (TPD): the higher the population density of a region, the more advanced innovation resources and factors will be correspondingly possessed, which can further realize the concentration of talents and the accumulation of knowledge, thus conducive to the improvement of regional innovation performance. In this paper, the number of permanent residents per square kilometer is used to measure the regional population density.

Seventh, the level of financial development (LFD): innovation activities need a large amount of financial support, its initial capital investment is large, long time to return, relying only on the main body of innovation expenditure can not meet the needs of innovation. Regional financial development can have an impact on regional innovation performance through corresponding financial support. This paper uses the proportion of deposits and loans in GDP of banking financial institutions to measure the level of regional financial development.

The data used in this paper’s study samples are from 31 Chinese provinces (cities and regions) between 2009 and 2020 (except from Taiwan, Hong Kong, and Macao Special Administrative Region), and China Science and Technology Statistical Yearbook, China Statistical Yearbook, Statistical Yearbook of Various Cities, and other sources provide the pertinent data, and interpolation is used to make up for individual missing data. The descriptive statistics for each variable (Table 1) are as follows.

**Table 1:** Descriptive statistics of variables.

VARIANT	SAMPLE SIZE	AVERAGE VALUE	(STATISTICS) STANDARD DEVIATION	MINIMUM VALUE	MAXIMUM VALUES
**RIP**	372	372	7.178	11.186	0.007
**ITA**	372	372	0.832	0.403	0.148
**LOI**	372	372	4.870	1.548	0.513
**TFI**	372	372	4.255	6.987	0 .0003
**LOU**	372	372	5.673	1.370	2.230
**TIL**	372	372	9.051	5.194	0.439
**TIS**	372	372	2.592	1.158	0.354
**TPD**	372	372	4.542	6.928	0.024
**LFD**	372	372	3.191	1.187	1.518

### 3.2. Modeling

#### 3.2.1. Spatial Durbin Model (SDM) construction

This research creates the Spatial Durbin Model (SDM), as illustrated below for discussion, in order to experimentally investigate the connection and spatial spillover impact of innovative talent aggregation on regional innovation performance:

$$RIP_{it}=\alpha_{0}+\alpha_{1}\sum_{j\neq i}^{n}W_{ij}RIP_{it}+\alpha_{2}\sum_{j\neq i}^{n}W_{ij}ITA_{it}+\alpha_{3}ITA_{it}+\alpha_{4}X_{it}++\eta_{it}+\varepsilon_{it}$$ (2)

The province’s regional achievement in innovation in year *t* is shown by *RIP*_*it*_, *ITA*_*it*_ shows the innovation talent aggregation of province *i* in year *t*, *X*_*it*_ shows the control variables of regional innovation performance, *α*_*1*_ is the estimated coefficient of spatial spillover of innovation performance of neighboring provinces, *α*_*2*_ describes the impact of innovation talent aggregation of neighboring provinces on the province’s present performance in terms of innovation, *α*_*3*_ describes the endogenous relationship of innovation performance of each province, *η*_*it*_ denotes time and province double fixed effects, *ε*_*it*_ is the model residual, Regions *i* and *j*’s distance is calculated using the *W*_*ij*_ spatial weight matrix. In this paper, two spatial weight matrices, geographic distance weight and economic geographic weight, are adopted to analyze.

#### 3.2.2. Construction of panel threshold models

In order to further explore the regulating mechanism of market and government on the aggregation of innovative talents and regional innovation performance, in this work, a panel threshold model is built using Eq (3):

$$\begin{array}{l} RIP_{it}=\beta_{0}+\beta_{1}ITA_{it}+\beta_{2}M_{it}+\beta_{3}ITA_{it}\times M\times I(M\leq n)\\ +\beta_{4}ITA_{it}\times M\times I(M>n)+\beta_{5}X_{it}+\eta_{it}+\varepsilon_{it} \end{array}$$ (3)

In Eq (3), the indicative function, denoted as *I (×)*, assumes a value of 1 when the condition enclosed in parenthesis is met and 0 otherwise, When *M* is less than or equal to *n* and larger than *n*, the coefficients of the threshold variable *M* are shown by the symbols *β*_*3*_ and *β*_*4*_, respectively, and the rest of the variables are the same as in the previous section.

## 4. Empirical analysis

As a population with higher human capital and diversity, talent has long-lasting impacts on regional socio-economic development via migration and redistribution [30]. The eastern coastal regions attracted a large pool of talent after 2000, while the central regions have encountered a severe brain drain dilemma. Western provinces such as Guangxi, Sichuan, Shaanxi and Gansu also had a negative migration efficiency, similar to the underdeveloped central regions. Other western provinces like Inner Mongolia, Chongqing, Guizhou, Yunnan, Tibet and Xinjiang had their migration efficiency breaking from negative to positive after 2000, indicating that these regions have attracted some talent to resettle, and it may be related to implementing regional development policies such as the China Western Development Plan. A considerable pool of talent from inland areas had migrated to very few eastern coastal regions after 2000, it also implies a concentration character of talent relocation.

In order to test the spatial correlation between innovative talent agglomeration and regional innovation performance, this paper firstly measured both global Moran’s I indexes between 2009–2020 to test the spatial correlation between innovative talent agglomeration and performance of regional innovation, as is seen from Table 2.

**Table 2:** Moran’s I Index from 2009 to 2020.

particular year	Geographic distance weighting		Economic geography weights	
	RIP	ITA	RIP	ITA
2009	0.093	0.411***	-0.073	-0.039
	(1.186)	(3.856)	(-1.408)	(-0.204)
2010	0.091	0.427***	-0.073	-0.039
	(1.150)	(3.968)	(-1.395)	(-0.184)
2011	0.103	0.449***	-0.069	-0.030
	(1.248)	(4.182)	(-1.242)	(0.118)
2012	0.135	0.391***	-0.064	-0.029
	(1.525)	(3.378)	(-1.076)	(0.141)
2013	0.156*	0.548***	-0.059	-0.010
	(1.701)	(4.943)	(-0.880)	(0.791)
2014	0.166*	0.516 ***	-0.055	-0.005
	(1.782)	(4.708)	(-0.751)	(0.962)
2015	0.217**	0.516 ***	-0.043	-0.002
	(2.222)	(4.734)	(-0.342)	(1.053)
2016	0.232**	0.536 ***	-0.039	-0.000
	(2.353)	(4.881)	(-0.204)	(1.126)
2017	0.174 *	0.531 ***	-0.049	-0.003
	(1.852)	(4.788)	(-0.544)	(1.039)
2018	0.171 *	0.519	-0.050	-0.011
	(1.837)	(4.680)	(-0.588)	(0.748)
2019	0.118	0.538***	-0.058	-0.006
	(1.378)	(4.876)	(-0.860)	(0.925)
2020	0.128	0.592***	-0.054	-0.008
	(1.447)	(5.314)	(-0.709)	(0.848)

Under the geographic distance weight matrix, regional innovation performance innovation talent aggregation shows an increasing trend with the growth of time, reaches the most significant in 2016, and then gradually decreases. Under the economic geography weight matrix, both regional innovation performance and innovation talent aggregation are insignificant and negative, gradually increasing. The gradual increase in the growth float over time shows that innovative talent aggregation possesses a noteworthy spatial correlation; the Moran index of regional achievements in innovation overwhelmingly passes the test, which shows that the improvement in the performance of regional innovation is not random, and will be affected by the spatial spillover from neighboring provinces. Regional innovation performance and innovation talent aggregation interact with each other in the development process, which can be analyzed using spatial econometric models.

### 4.1. Constructing a spatial Durbin model

This research explains how inventive talent clustering affects regional performance in innovation using the spatial Durbin model with double fixed impacts in time and location, as seen in the table’s regression analysis of the effect of creative talent clustering on regional achievement in innovation. The impact coefficient under the full sample is, indicating that at the 5% level innovative talent agglomeration plays a positive influence effect on the enhancement of regional achievements in innovation, and demonstrates a significant spatial spillover impact. As the creator and participant of innovation, the agglomeration of innovative talents in a certain region can promote the degree of technological innovation, enhance the level of knowledge spillover, and have a certain radial pull effect on the surrounding region. With higher mobility, interaction, and competition in the contemporary world, attracting and retaining highly skilled talent has become essential for countries and regions [31]. With the introduction and cultivation of talents, it is conducive to the updating of new technologies, new processes and new products, while increasing knowledge sharing to lessen the obstacles caused by information asymmetry, and enhancing the degree of specialization through different forms of exchanges and seminars, which demonstrates the diversification of knowledge spillovers and then promotes the output of regional achievements in innovation. Table 3 below displays the outcomes:

**Table 3:** Regression results based on SDM.

variant	Geographic distance weighting	Economic geography weights
WRIP	-0.304***	-0.341
	(-4.20)	(-1.37)
WITA	3.030	24.274
	(0.69)	(1.41)
ITA	-1.980	2.852
	(-1.04)	(1.29)
LOI	-0.121**	-0.122*
	(-2.60)	(-2.57)
TFI	0.205*	0.253**
	(2.57)	(2.63)
LOU	-0.912***	-0.269
	(-4.58)	(-1.40)
TIL	-0.041	-0.071
	(-1.14)	(-1.80)
TPD	0.034**	0.043***
	(3.04)	(4.17)
LFD	-0.021*	-0.050***
	(-2.57)	(-5.62)
TIS	-0.107	0.054
	(-1.73)	(0.82)
R^2^	0.172	0.137
N	372	372

The estimation of the spatial lag term under the double fixed-effects model with geographic weighting matrix shows that: (1) the innovation performance of neighboring provinces significantly inhibits the gathering of innovative talents in this province (-3.04***), and (2) the gathering of innovative talents in neighboring provinces promotes the innovation performance of this province (3.030), but it is not significant. (3) Agglomeration of innovative talents in the province will inhibit the innovation performance of the province (-1.980). Analyzing the results of the estimate of the control variables, it is evident that foreign investment, population density will significantly promote the innovation performance of this province at the 10% and 5% levels, while the rest of the control variables are able to enhance the regional performance of innovation to varying degrees, among which the urbanization degree variable has the most obvious inhibitory effect.

According to the weight matrix for economy and geography, the estimation results of the spatial lag term show that:

(1) the innovation performance of neighboring provinces will inhibit the innovation performance of this province (-0.341), but it is not significant, which indicates that to some degree the region can produce the effect of enhancing the innovation performance through the pooling of talents;

(2) The innovative talents gathering in neighboring provinces will encourage this province’s innovative performance (24.274), but it is not significant, indicating that there exists a certain degree of independence among provinces, and the sharing of knowledge workers and exchange of talents have not reached the situation of complete information symmetry;

(3) The innovative talents gathering in this province will promote the innovation performance of this province (2.852), which shows that the significant level is not significant, indicating that the factors affecting the innovation performance of this province are not only the talent gathering, but also affected by a variety of other factors, and it is necessary to include the control variables for deeper estimation.

Additionally, the direct, indirect, and total impacts of this paper’s SDM are broken down as indicated in Table 4 below:

**Table 4:** Direct, indirect and total effects.

variant	Geographic distance weighting matrix			Economic geography weighting matrix		
	direct effect	indirect effect	aggregate effect	direct effect	indirect effect	aggregate effect
ITA	-2.193	2.846	0.653	2.612	17.842	20.454
	(-1.12)	(0.83)	(0.19)	(1.18)	(1.37)	(1.50)
LOI	-0.120***	-0.060	-1.80**	-0.119***	-0.589	-0.708
	(-3.01)	(-0.69)	(-2.09)	(-3.05)	(-1.25)	(-1.50)
TFI	0.193*	0.230	0.423***	0.254**	0.545	0.799
	(2.14)	(1.46)	(2.82)	(2.59)	(0.59)	(0.83)
LOU	-0.907***	0.160	-0.747***	-0.350*	6.755***	6.405***
	(-3.98)	(0.49)	(-3.08)	(-1.70)	(3.81)	(3.47)
TIL	-0.093**	0.585***	0.492***	-0.074*	-0.365	-0.439
	(-2.00)	(7.64)	(6.89)	(-1.66)	(-1.26)	(-1.43)
TPD	0.032**	0.034	0.066***	0.047***	-0.241*	-0.194
	(2.59)	(162)	(4.69)	(4.72)	(-1.78)	(-1.39)
LFD	-0.021***	0.007	-0.014	-0.048***	-0.109	-0.157**
	(-2.71)	(0.55)	(-1.11)	(-5.75)	(-1.61)	(-2.26)
TIS	-0.124**	0.094	-0.030	0.018	1.548***	1.566***
	(-2.19)	(0.82)	(-0.24)	(0.31)	(3.32)	(3.27)

As can be seen from Table 4 above, first of all, except for the degree of financial development and urbanization rate, other control variables are all smaller than the weight of economic geography under the weight of geographical distance, which means that with the development of economy, the direct effect of foreign investment has a stronger role in promoting regional innovation performance.

Secondly, the significance of the indirect effect of urbanization level, population density and investment scale is stronger under the weight of economic geography than under the weight of geographical distance, while the indirect effect of transportation infrastructure construction is the opposite, which means that the widening of economic gap, the indirect effect of urbanization level and investment scale can promote the development of regional innovation performance. The indirect effect of transportation infrastructure construction on regional innovation performance weakened, and the indirect effect of population density inhibited the development of regional innovation scale.

Finally, the significance of the total effect of informatization degree, transportation infrastructure construction and population density is lower in the economic geography weight matrix than in the geographical distance weight matrix, and the significance of the total effect of other variables is on the contrary, which means that with the widening of economic gap, the total effect of informatization degree has a weaker inhibitory effect on regional innovation performance. The total effect of financial development on regional innovation performance was strengthened. So H1 is true.

### 4.2. Threshold identification and threshold effect analysis

In this paper, utilizing the Bootstrap repeated sampling technique as the threshold variables, the threshold features of government assistance and marketization level are investigated, and the samples are repeated 500 times, as shown in Table 5 Threshold Characteristics Estimation Results:

**Table 5:** Threshold characteristics estimation results.

Threshold variables	single threshold			double threshold			triple threshold		
	Estimated threshold	F-value	P-value	Estimated threshold	F-value	P-value	Estimated threshold	F-value	P-value
Marketization Level	37.095	*18*.*08*	*0*.*203*	51.344	11.54	0.477	52.101	*10*.*96*	*0*.*613*
government support	36.643	*132*.*33*	*0*.*000*	60.285	11.73	0.074	75.536	*3*.*32*	*0*.*786*

It can be seen in Table 5 that there is no threshold feature between marketization level and regional innovation performance. Based on the threshold feature estimation, this paper uses the panel threshold model to investigate the reasonable interval of the adjustment mechanism between marketization level and government support on the relationship between innovation talent agglomeration and regional innovation performance growth. None of the P-values in the range of 5% passed the significance test. After the marketization level crossed the threshold, the improvement effect of innovation talent agglomeration on regional innovation performance was not affected by the degree of marketization, and the moderating effect was not significant, that is, hypothesis H3 was established. The possible reason for the absence of threshold characteristics is that innovation entities need a large amount of financial support in the process of R&D. Compared with the process of marketization, talent agglomeration is more concerned about factors such as local R&D funding input or material incentives. Although the deepening of marketization is conducive to the stability of the regional price mechanism, However, there is still a disconnect between the technical problems in research and innovation and the market demand.

**Fig 2:**
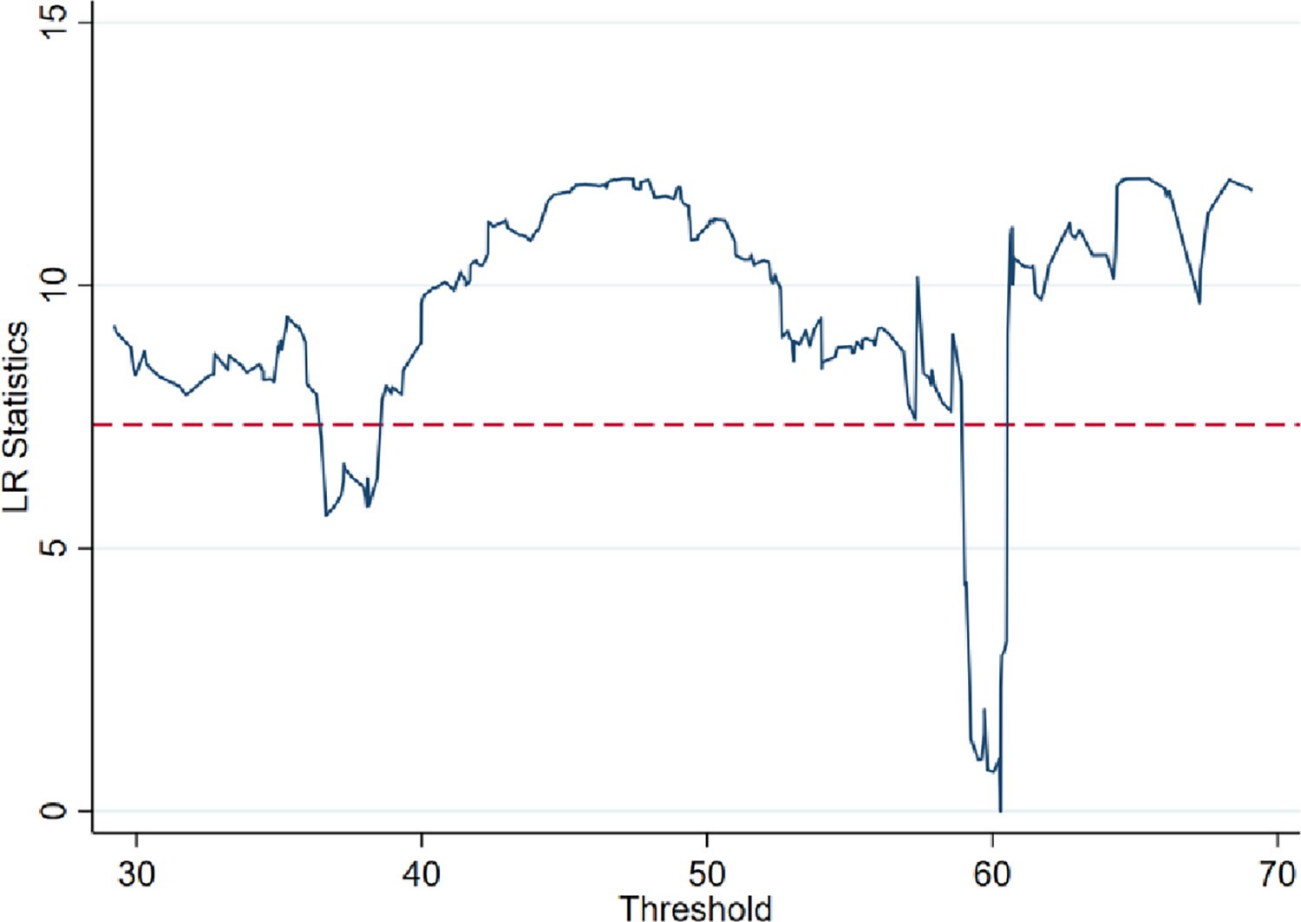
Double threshold effect.

In addition, the single threshold of government support is significant at the level of 1%, the double threshold is significant at the level of 10%, and the three thresholds are not significant and do not exist, indicating that the moderating effect of government support on the concentration of innovative talents and regional innovation performance has a double threshold effect, as shown in the Fig 2 below.

After performing threshold identification the dual threshold model is selected for testing the threshold regression and Table 6 demonstrates the results of the regression on the panel threshold model.

**Table 6:** Threshold regression parameter results.

variant	market-oriented	government support
LOI	-0.066	-0.012
	(-0.81)	(-0.17)
TFI	0.251**	0.205 ***
	(2.70)	(2.73)
LOU	0.588**	0.577 **
	(2.06)	(2.52)
TIL	0.049	-0.005
	(0.62)	(-0.09)
TPD	0.103**	0.046
	(2.28)	(1.48)
LFD	-0.010	-0.012*
	(-1.29)	(-1.78)
TIS	0.471	-0.020
	(0.46)	(-0.21)
Interval one		3.539***
		(1.41)
Interval two		0.86*
		(0.38)
Interval three		3.648***
		(1.21)
R^2^	0.996	0.986

After adding the threshold variable of government support, the affect of innovative talent agglomeration on the growth rate of regional achievements in innovation shows a double threshold effect with the enhancement of urban areas’ potential for innovation. The regional achievements in innovation can be divided into influence intervals according to its threshold value: the first interval (η≤36.6643), the second interval (36.643≤η≤60.285), and the third interval (η≥75.536), when the regional innovation capability is in the first interval (η≤36.6430), for every 1% increase in the pooling of innovative talents, the city’s economy develops at a higher, more superior level by 3.539%, when the regional innovation capacity is in the second interval (36.643≤η≤60.285), an increase of 1% in creative talent agglomeration results in a 0.86% improvement in the economy’s superior growth level, and when the regional innovation capacity is in the third interval (η≥60.285), the economy will see a 3.648% rise in superior growth for every 1% increase in creative talent agglomeration. With the increase of government support, the rate of increase of innovative talent agglomeration for regional innovation performance has a marginal contribution that decreases first and then increases, which verifies hypothesis H3 is true. The reason for this is that the R&D funding given by the government can alleviate the funding gap of basic research topics such as the development of common knowledge to a certain extent, but it is yet to be known whether the funding distribution is reasonable and whether the funding support is able to satisfy the innovation needs of the region where it is located. The government can stimulate individual innovation initiative by playing an effective regulatory tool, but the irrational or wasteful use of financial resources and imbalance in distribution will also weaken the role of positive regulation. In the continuous exploration and optimization of the forward, fully stimulate the enthusiasm of talent creation, so as to drive the improvement of regional achievements in innovation.

## 5. Conclusions and recommendations

Explore the spatial effect between the innovative talent agglomeration brought by the innovation ability on the development of regional achievements in innovation, analyze the process of spatial spillover effect of China’s innovative talent agglomeration on regional achievements in innovation in the past twelve years, comprehensively evaluating the antecedents and consequences of the two and the degree of correlation, verifying the threshold identification and effect of innovative talent and regional achievements in innovation under the regulation of the level of marketization, government support, and the following are the study’s findings:

Firstly, as seen from the standpoint of time series evolution, China’s innovative talent agglomeration fish innovation performance output there is a certain degree of correlation, talent agglomeration to achieve a gradual coastal transfer, and by the higher economic level of the region tends to transfer to the surrounding provinces of the tendency to slow down to a certain extent the asymmetric phenomenon of information brought about by the resource grabbing to avoid the emergence of talent crowding phenomenon, the innovation performance output of the twelve-year period demonstrated a steady The innovation performance output has shown a steady development trend in the past twelve years.

Secondly, as seen from the standpoint of spatial pattern distribution, the radiation effect of the eastern and coastal regions as the leading role is still maintained, and the talent power of remote areas such as Xinjiang and Tibet is thin, and the innovation performance output is in a disadvantageous state, but it is also slightly improved in terms of itself. Most of the innovative talents are concentrated in the north, Shanghai, Guangzhou and coastal economically developed regions, and show the spatial spillover effect.

Thirdly, from the perspective of transmission path, the degree of marketization and the strength of government support are used as moderating variables for threshold identification and effect analysis, which verifies the existence of a twofold threshold result of government support between the concentration of innovative talents and the regional innovation performance, and confirms the validity and safeguard effect of government support. These conclusions also illustrate that government support has an important guiding role in the enhancement of long-term innovation performance, and government support further broadens the research system of innovation talent agglomeration effect and the application boundaries of the theory of innovation talent resource allocation. In light of this, the next suggestions are made:

(1) Firm innovation-driven orientation within each region, configure regional innovation resources according to local conditions, rationally lay out the industrial structure, strengthen cooperation and exchanges between industries, research facilities and universities, and rationally configure the distribution of spatial patterns of innovation talent concentration. According to the division of industrial clusters in different regions, provide matching degree for innovative talents and industrial clusters, optimize the allocation efficiency of innovative talents, improve the degree of fit between counterpart institutions and the introduction of talents, promote the output of innovation performance in the region, and raise the area of poorer quality economic development’s marginal contribution from the concentration of creative talent. Reasonable layout of primary, secondary and tertiary industries, optimization and upgrading of industrial structure, complementing strengths and weaknesses, rational layout, accelerating urban-rural integration, and providing hardware conditions for scientific and technological talent clustering with geographical advantages.

(2) Firm the cultural identity and cultural self-confidence of innovative talents, actively guide the government’s synergistic allocation of innovative talents’ resources, digital transformation and regional innovative talents’ development, and stimulate the development momentum of innovative talents’ new business models in the field of science and innovation. The government’s management should plan the number of innovative talents in its region, play the capacity of macro-control and organization and coordination, and reduce the lack of information brought by information asymmetry to the main body of innovation through knowledge sharing, information disclosure, and exchange and communication. In terms of subsidies and other material incentives, it should strive to allocate limited research funds to support basic science research talent funding subsidies, and withdraw from the more applied and competitive areas of technological innovation.

(3) Strengthen government management, improve government service functions, optimize the allocation structure of government R&D funds, enhance the efficiency of fund allocation, and observe the preferences of creative individuals, so as to avoid the excessive flow of talents to economically developed regions while neglecting the advancement of innovative science and technology in the less developed regions. When the government’s overall planning, it should consider the improvement of education, medical care, transportation infrastructure construction and other guaranteed resources as appropriate, improve the ecological environment for the introduction of higher education talents, inspire creative individuals to go back to their hometowns and construct, and reduce the outflow of talents; at the same time, the government encourages the establishment of sub-campuses of higher education institutions in small regions with closer geographic distances, to reduce the phenomenon of talent congestion, increase local employment opportunities, and realize the regional high-quality development. A win-win situation.
